# The integrated screening action model (I-SAM): A theory-based approach to inform intervention development

**DOI:** 10.1016/j.pmedr.2021.101427

**Published:** 2021-05-31

**Authors:** Kathryn A. Robb

**Affiliations:** Institute of Health and Wellbeing, University of Glasgow, Glasgow G12 0XH, United Kingdom

**Keywords:** Screening, Theory-based, Model, Framework, Intervention development, Cancer, Diabetic retinopathy, Abdominal aortic aneurysm

## Abstract

Screening can reduce deaths if the people invited participate. However, good uptake is hard to achieve, and our current approaches are failing to engage the most vulnerable. A coherent model of screening behaviour to guide our understanding and intervention development is yet to be established. The present aim was to propose an Integrated Screening Action Model (I-SAM) to improve screening access.

The I-SAM synthesises existing models of health behaviour and empirical evidence. The I-SAM was developed following: i) an appraisal of the predominant models used within the screening literature; ii) the integration of the latest knowledge on behaviour change; with iii) the empirical literature, to inform the development of a theory-based approach to intervention development.

There are three key aspects to the I-SAM: i) a sequence of stages that people pass through in engaging in screening behaviour (based on the Precaution Adoption Process Model); ii) screening behaviour is shaped by the interaction between participant and environmental influences (drawing from the Access Framework); and iii) targets for intervention should focus on the sources of behaviour - ‘capability’, ‘opportunity’, and ‘motivation’ (based on the COM-B Model).

The I-SAM proposes an integrated model to support our understanding of screening behaviour and to identify targets for intervention. It will be an iterative process to test and refine the I-SAM and establish its value in supporting effective interventions to improve screening for all.

## Highlights

• Screening is a major public health strategy in disease prevention.
• Uptake remains suboptimal and current approaches fail to engage the most vulnerable.
• Interventions to improve equitable access are urgently required.
• A coherent model of screening behaviour is yet to be established.
• The Integrated Screening Action Model (I-SAM) proposes an integrated theoretical model aiming to improve screening access for all.

## 1 Introduction

**Fig. 1:**
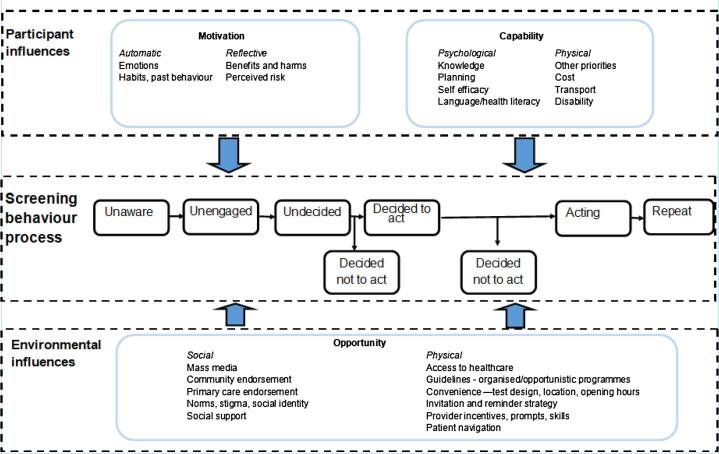
Integrated Screening Action Model (I-SAM).

Screening can reduce deaths if the people invited participate (Ronco et al., 2014, Myers et al., 2015, National Lung Screening Trial Research Team, 2011, Lin et al., 2016, Guirguis-Blake et al., 2019, Leese et al., 2015). Future technological advances will lead to more accurate, and stratified screening tests, offering improvements in early diagnosis and survival. However, no matter how state-of-the-art the test, it will only be effective if people are willing to do it. Years of experience of cancer screening suggest that good uptake is hard to achieve, and our current approaches fail to engage the most vulnerable (McCowan et al., 2019). The existing literature on screening behaviour while informed, in some cases, by theory, has not yet established a coherent action model of screening behaviour to guide our understanding of the determinants of screening behaviour and identify targets for intervention (Rakowski and Breslau, 2004, Kobrin et al., 2015). The aim of this paper is to describe the development of an Integrated Screening Action Model (I-SAM: Fig. 1), which draws on theoretical models from behavioural science and empirical evidence, to provide a unifying structure to improve the translation of research into practice to increase the efficacy of existing and future screening tests.

Participation in screening programmes (breast, colorectal, cervical, lung, diabetic retinopathy, abdominal aortic aneurysm) remains suboptimal, with persistent inequalities in uptake such that people living in more socioeconomically deprived areas, ethnic minorities, people with comorbidities, and people with intellectual disabilities are less likely to participate (Campbell et al., 2020, McCowan et al., 2019, Szczepura et al., 2008, Crilly et al., 2015, Leese et al., 2008). There is an urgent need to improve screening participation and develop effective interventions (Duffy et al., 2017).

One of the key principles of intervention development is that it should draw on existing theory (O’Cathain et al., 2019). A theoretical model can help to guide intervention research from conceptualisation to analysis and clarify why, how, and for whom an intervention may work (Kobrin et al., 2015). To date, researchers have drawn on a range of health behaviour models and theories to study screening behaviour yet despite calls for the need for multilevel theoretical and conceptual approaches (Rakowski and Breslau, 2004, Curry and Emmons, 1994) little progress has been made.

## 2 Approach to model development

The development of the I-SAM was guided by three key principles: i) the model should account for people being at different stages of the screening behaviour process i.e. some people are unaware, some people have formed an intention to screen; ii) screening behaviour is influenced at multiple, interacting levels; and iii) existing models of behaviour change and empirical evidence should inform the development of the I-SAM. Following these three principles, the application of the I-SAM should improve the prediction of screening behaviour and support the identification of intervention targets to enhance the screening process.

The I-SAM was developed following: i) an appraisal of the predominant models used within the screening literature; ii) integration of the latest knowledge on behaviour change; with iii) the empirical literature to inform the development of a theory-based approach to intervention development. Following the approach of von Wagner et al., (2011), the aim was not to conduct a systematic review of the diverse literature on the use of theory or constructs related to screening access (e.g. Cooke and French, 2008, Priaulx et al., 2020, Graham-Rowe et al., 2018, Sabatino et al., 2012), but to provide a novel synthesis of the theoretical and empirical literature to inform researchers and practitioners tasked with improving access to screening. Theories and models are well established in the behavioural science literature but have not been applied extensively to the screening context nor integrated with the empirical literature. This approach therefore aimed to bring together in a unifying structure the state of research to aid intervention design, outcome measures and process evaluation (Redman et al., 2015).

The predominant models used within screening research have been in the context of cancer screening and reflect the key models within Health Psychology (Table 1). These same models broadly align with the use of health behaviour theory in cancer screening in funded grant applications (Kobrin et al., 2015). There are merits in each of the existing models of health behaviour identified in Table 1. Other models and frameworks have also been used, to a lesser extent, in studying screening behaviour e.g. Attitude Social Influence Self-efficacy Model (Vries and Mudde, 1998), Preventive Health Model (Watts et al., 2003), Psychosocial Determinants of Socioeconomic Inequalities in Cancer Screening (von Wagner et al., 2011), Theoretical Domains Framework (Graham-Rowe et al., 2018). However, it is clear from Table 1 that no single model is routinely used in screening behaviour research, and typically, researchers incorporate elements from different models (e.g. Kobrin et al., 2015). This approach of incorporating components from different models can work well for those fluent in health behaviour models and screening research, but can be ad hoc, and it can be challenging for health professionals with little background in behaviour change models. Therefore, the aim of proposing a new integrated model was to build on and synthesise the key components of existing models of health behaviour and empirical evidence, to develop a parsimonious model of screening behaviour that would be helpful to those wishing to understand screening behaviour and how to intervene.

**Table 1:** Selected predominant models used in screening research.

Model	Basic premise	Example studies
Health Belief Model (Rosenstock, 1974)	Behaviour result of beliefs about: perceived susceptibility; perceived severity; benefits and barriers; cues to action	Wardle et al., 2000, Yarbrough and Braden, 2001, Orbell et al., 1996
Theory of Reasoned Action (Ajzen and Fishbein, 1980), Theory of Planned Behaviour (Ajzen, 1991)	Behaviour result of attitudes, subjective norm, and perceived behavioural control* predicting intention and then behaviour. *can directly impact behaviour	Cooke and French, 2008, Orbell et al., 2006, Drossaert et al., 2003, Rutter, 2000, Devellis et al., 1990
Protection Motivation Theory (Rogers, 1975)	Behaviour determined by threat appraisal and coping appraisal including key components of: perceived severity; perceived susceptibility; response efficacy and self-efficacy	Orbell and Sheeran, 1998, Li et al., 2020
Precaution adoption process model (Weinstein and Sandman, 1992)	Stage model explaining how a person decides to take action and how that decision translates into action	Costanza et al., 2005, Costanza et al., 2009, Ferrer et al., 2011, Marlow et al., 2018
Transtheoretical Model (Prochaska and DiClemente, 1982)	Stage model synthesising 18 therapies to elicit and maintain behaviour change. Key stages include: pre-contemplation; contemplation; preparation; action; maintenance	Rakowski et al., 1996, Lipkus et al., 1996, Trauth et al., 2003, Kelaher et al., 1999
Social Cognitive Theory (Bandura, 1986)	An extension of Social Learning Theory proposing a dynamic and reciprocal interaction of the person, environment, and behaviour. Key components include: outcome expectancies; reciprocal determinism; behavioural capacity; modelling; social reinforcement; self-efficacy	Suarez et al., 1993, Braun et al., 2005

## 3 The integrated screening action model (I-SAM)

The I-SAM (Fig. 1) is an integrated and theoretically informed model to support our understanding of screening behaviour and identify targets to increase access to screening. There are three key aspects to the I-SAM: i) a progressive sequence of stages that people pass through in engaging in screening behaviour; ii) screening behaviour is shaped by the synergistic interaction between participant and environmental influences; iii) targets for intervention should focus on the sources of behaviour including ‘capability’, ‘opportunity’, and ‘motivation.’ Because the I-SAM integrates existing models of behaviour and behaviour change and empirical evidence, it begins with some supporting evidence for their potential utility.

### 3.1 Screening behaviour process

The central component of the I-SAM, the *Screening behaviour process,* is based on the Precaution Adoption Process Model (PAPM; Weinstein et al., 2008). The PAPM identifies seven stages in the process of precaution adoption and was initially applied to home radon testing (Weinstein and Sandman, 1992). Just as some diseases such as cancer can develop sequentially (e.g. the colorectal adenoma carcinoma sequence, Leslie et al., 2002) so too can screening behaviour, developing through a sequence of stages or steps. Dividing screening behaviour development into distinct stages is helpful in identifying stage-specific targets for intervention, again in a similar manner to how cancer itself can be targeted based on stage e.g. premalignant vs. metastatic. The PAPM therefore offers a framework for understanding screening behaviour that can be readily understood from multidisciplinary perspectives.

Stage-model approaches are advantageous because they are easily understood and they acknowledge that a ‘one-size-fits-all’ approach may have limitations (Ferrer et al., 2011); although one stage model, the Transtheoretical Model (Table 1; Prochaska and Diclemente, 1982), has attracted considerable criticism (West, 2005). A stage model offers the opportunity to target interventions to different sectors of the community based on a population’s readiness to engage with screening behaviour e.g. people living in socioeconomic deprivation, ethnic minorities, and people with comorbidities and intellectual disabilities. A more targeted approach can therefore better address inequalities in access and ensures the benefits of screening can be fully realized by all in society. More broadly, this targeted approach aligns with the concepts of proportionate universalism (Marmot and Bell, 2012) and precision medicine (Hekler et al., 2020) that recognize the need to tailor interventions based on people’s need.

The PAPM (Weinstein et al., 2008) describes the stages defined as psychological processes that people pass through in precaution adoption from ‘unaware’ to ‘unengaged’ to ‘deciding’ to ‘intending’ to ‘acting’ to ‘repeat’ (Fig. 1). Table 2 describes the various stages using colorectal cancer screening as an example. Most predominant models used within screening research (Table 1) focus on how people who get to the decision making (undecided) stage, decide what to do. However, this is to the detriment of those who fail to reach that stage (~30% in survey samples: Ferrer et al., 2011, Costanza et al., 2005) and emphasises the value in including the unaware and unengaged stages. Within behavioural science the intention-action gap is well-recognised (Orbell and Sheeran, 1998, Gollwitzer and Sheeran, 2006), and so the I-SAM acknowledges that not everyone will progress from *decided to act* to *acting* resulting in people joining the *decided not to act* stage. The *decided not to act stage* can be further broken down in to disinclined abstainers (people who are not inclined to screen and don’t) and inclined abstainers (people who are inclined to screen but fail to act), with the latter group a particularly important group when considering improving access to screening (Orbell and Sheeran, 1998, Power et al., 2008). For those who complete screening it may be necessary to attend subsequent follow up tests (e.g. colposcopy for cervical screening), which are not elaborated here. There is also the option, depending on the type of screening, to *repeat* screening when next invited (Table 2). Including the *repeat* stage in the I-SAM is important because many models focus on the initiation of behaviour (e.g. Table 1) rather than maintenance, and while screening is an infrequent behaviour, repeated screening behaviour is necessary but remains relatively understudied (Lo et al., 2015). Adopting a behaviour for the first time is different to repeating the behaviour (Weinstein et al., 2008), and intervention approaches need to reflect this.

**Table 2:** Screening behaviour stages for a colorectal cancer screening example.

	Colorectal screening example
Unaware	Never heard of colorectal screening
Unengaged	Never thought about colorectal screening
Undecided*	Undecided about colorectal screening
Decided to act*	Decided to colorectal screen
Acting	Completing colorectal screening test
Repeat	Complete colorectal screening when next invited
*Decided not to act	Decide not to colorectal screen

Conceptualising screening behaviour in these seven stages permits the identification of distinct groups of people who may require tailored interventions to improve screening access. Weinstein et al. (1998) propose that stage theories have four key elements and assumptions. Firstly, the stages represent an ideal or ‘prototype’ to assist with intervention development. In reality, there may be overlap between stages. Secondly, stage theories assume that people progress through a sequence of stages. However, people may not progress, they may regress, or they may progress so rapidly they can be viewed as skipping stages e.g. if a woman is offered cervical screening while attending primary care for another reason, she may progress from unengaged to action with little deliberation. Thirdly, people in the same stage will face common barriers and so targeting interventions to stage can assist in supporting people to progress to the next stage. Fourthly, people in different stages will face different barriers requiring interventions targeted to their barrier and stage.

Several studies have already illustrated the value of using the Precaution Adoption Process Model to identify people at different stages in the screening process for breast (Costanza et al., 2009), cervical (Marlow et al., 2018), and colorectal (Costanza et al., 2005, Ferrer et al., 2011) screening, and that health beliefs differ across stages. The next step for research is to develop interventions to target these beliefs at the various stages.

### 3.2 Participant and environmental influences

Within the I-SAM, the dual impact of *Participant* and *Environmental* influences synergistically shape the central *Screening behaviour process.* There are multiple levels of influence on screening behaviour (Priaulx et al., 2020), and these were well-described by Taplin et al. (2012) in their description of the seven levels of influence in cancer. The I-SAM takes a more parsimonious approach with two overarching levels: participant and environmental influences. This approach of incorporating both the *Participant* and *Environmental influences* aligns with the Access Framework’s demand- and supply-side determinants (Richard et al., 2016). The Access Framework is from the Primary Care literature and has not yet been applied to screening behaviour. By simultaneously considering both *Environmental* (how and where screening is offered) and *Participant* (people’s willingness and ability to engage with screening) influences, the I-SAM provides a rigorous structure to understand the interdependent influences of environmental and participant factors in screening access. The predominant models used within screening research (Table 1) have typically focused more on participant influences on screening behaviour to the neglect of environmental influences, with the exception of Social Cognitive Theory (Bandura, 1986). By considering the *Participant* and *Environmental* influences simultaneously, this will more rapidly produce improvements in access. Marteau and colleagues powerfully argue for applying psychological evidence to the shaping of *Environmental influences* (e.g. ease of effort, product design) and suggest this approach has greater potential to impact behaviour than interventions encouraging people to reflect on their behaviour – the *Participant influences* side (Marteau et al., 2012). Furthermore, simulation model research also suggests that participant focused interventions alone are less effective than using environmental or a combination of participant and environmental interventions (Hosking et al., 2013).

### 3.3 Sources of behaviour: ‘capability’, ‘opportunity’, and ‘motivation’

The third component of the I-SAM draws on the COM-B Model (Michie, Stralen and West, 2011) which identifies the sources of behaviour that can be targets for interventions. The COM-B is widely used to assist behaviour change intervention developers to identify what needs to change for interventions to be effective, yet few studies have used it to support screening research (e.g. Rogers et al., 2019, Kerrison et al., 2018). The COM-B Model suggests that behaviour can be understood in terms of ‘capability’, ‘opportunity’, and ‘motivation’ and interventions need to change one or more of these constructs to effectively support screening behaviour. Within the I-SAM, ‘capability’ and ‘motivation’ have been conceived as relating to *Participant influences* while ‘opportunity’ relates more to *Environmental influences*, however in line with the COM-B Model, it is recognized that behaviour is part of an interacting system so that increasing capability or opportunity can also increase motivation (West et al., 2020).

The I-SAM also contains within ‘capability’, ‘opportunity’, and ‘motivation’ suggested targets for future interventions based on the empirical screening literature. The COM-B Model specifies that ‘motivation’ comprises both automatic motivation and reflective motivation – in line with Dual Process Theory (Kahneman, 2011, Strack and Deutsch, 2004). Within the I-SAM, automatic motivation includes negative emotional responses to screening such as fear, fatalism, disgust, embarrassment (Kotzur et al., 2020, Sarma et al., 2019, Wardle et al., 2015, Piyasena et al., 2019) as well as habits and past behaviours such as previous experience of screening and tendency to follow health recommendations. Reflective motivation involves conscious evaluations such as evaluation of the benefits and harms of screening (Hall et al., 2015, Wardle et al., 2015, Piyasena et al., 2019, Ahmad et al., 2020), and perceived risk (Katapodi et al., 2004, Vernon, 1999, Ferrer et al., 2016). There may be overlap in the extent to which motivations are automatic or reflexive. For example, emotions may be automatic in terms of a physiological fear response to the word ‘cancer’ while also eliciting more reflective thinking on the fear of cancer.

‘Capability’ comprises both psychological and physical skills to enable screening behaviour. Psychological capability for screening includes having the cognitive resources to undertake the processes involved in completing screening which could include planning where, when and how you will complete a home-based test or planning and arranging an appointment for a clinic-based test and working out how to get there (Kotzur et al., 2020). Psychological capability includes self-efficacy – the belief that you can do the action required – which is a fundamental component of behaviour change (Bandura, 1986) and has been found to influence screening behaviour (Cooke and French, 2008, Duncan et al., 2014). Psychological capability also includes having language and health literacy skills to engage with screening (Graham-Rowe et al., 2018, van Allen et al., 2021, von Wagner et al., 2009). Physical capability to perform screening includes people having other priorities (e.g. comorbidities, family responsibilities) which limits their capability to engage with screening (McCowan et al., 2019, Hall et al., 2015, Kotzur et al., 2020, Graham-Rowe et al., 2018). A person may be unable to access screening due to the financial costs of taking time off work or travelling to a screening clinic (Brown et al., 2000, Sabatino et al., 2012, Graham-Rowe et al., 2018, Ahmad et al., 2020). Physical capability also relates to disabilities, which may impede screening e.g. visual impairment may impact on self-completed screening tests while reduced mobility may impact on attending clinic-based screening (Kotzur et al., 2020).

‘Opportunity’ includes both the social opportunity and the physical opportunity and the existing literature points to several potential targets to increase access to screening in both. Social opportunity includes social cues in the environment which can influence screening behaviour such as mass media (Marlow et al., 2012, Casey et al., 2013, Macdonald et al., 2018, Durkin et al., 2019, Graham-Rowe et al., 2018), community endorsement (Martini et al., 2016, Larkey, 2006, Graham-Rowe et al., 2018), primary care endorsement (Wardle et al., 2016, Duffy et al., 2017), norms, stigma and social identify (Sieverding et al., 2010, Smith-McLallen and Fishbein, 2008, Lo et al., 2015b, Vrinten et al., 2019, Jetten et al., 2017), and social support (Katapodi et al., 2002, Larkey, 2006, Documet et al., 2015, Graham-Rowe et al., 2018). Physical opportunity relates to aspects of the physical environment which influence the opportunity to access screening such as access to healthcare and healthcare insurance (Power et al., 2009, Taplin et al., 2012, Graham-Rowe et al., 2018, Piyasena et al., 2019, Bird and Davis, 2015), whether national guidelines recommend screening, and if screening is offered as part of an organized or opportunistic programme (Wardle et al., 2015, Miles et al., 2004, Graham-Rowe et al., 2018). Convenience can also influence the physical opportunity to access screening including design of the test – such that the easier the test is to do, the more likely people are to do it, location of screening (e.g. rurality, access to public transport), opening hours, waiting time on day of appointment, one-stop-shops and side effects (Robb and O'Carroll, 2019, Sabatino et al., 2012, Graham-Rowe et al., 2018, Piyasena et al., 2019, Cavan et al., 2017, van Allen et al., 2021, Hipwell et al., 2014). Opportunity is further influenced by the invitation and reminder strategy offered by the screening provider (Duffy et al., 2017, Graham-Rowe et al., 2018, Hipwell et al., 2014, Chaudhry et al., 2012), and whether providers are incentivized or receive prompts or skills training to engage people in screening (Sabatino et al., 2012, Brouwers et al., 2011). Physical opportunity to access screening can also be influenced by the availability of patient navigators to support people through the screening process (Jandorf et al., 2005, Robinson-White et al., 2010).

The additional benefit of including the COM-B model within the I-SAM is that it forms the central hub of the broader Behaviour Change Wheel (Michie et al., 2011). Surrounding the hub is a layer of nine intervention functions (education, persuasion, incentivisation, coercion, training, enablement, modelling, environmental restructuring, restrictions) which can be used to support screening behaviour. The outer layer relates to seven policy categories (environmental/social planning, communication/marketing, legislation, service provision, regulation, fiscal measures, guidelines) that can support the delivery of these interventions.

## 4 Using the I-SAM to improve access to screening

An illustration of how the components of the I-SAM can be used to identify and target interventions to improve access to screening is provided in Table 3. Table 3 maps the central Screening Behaviour Process (column 1) with intervention targets derived from the COM-B and the empirical literature (column 2) with intervention functions (column 3) and policy categories (column 4) taken from the Behaviour Change Wheel to improve access to screening. Table 3 describes the various stages within the screening behaviour process and elaborates the key targets within the components of the COM-B with intervention and policy solutions. Different interventions will be required based on where someone is in the screening process. For example, among people who are unaware, unengaged, or undecided, an awareness raising campaign addressing the benefits and harms of screening could be a motivational target. Among people who have decided to act, supporting people to make a plan about how, when and where they will do screening offers a capability target.

**Table 3:** Illustration of how the I-SAM components identify potential targets and policies to increase access to screening.

Screening behaviour process	Intervention targets	Intervention function	Policy
**Preintention** Unaware Unengaged Undecided	**Participant influences** ***Motivation*** Knowledge of benefits and harms Perceived risk Emotions Identity ***Capability*** Self-efficacy, cost, transport, disability	Education, persuasion Enablement	**Communication/marketing** Awareness raising campaign addressing motivational factors Awareness raising campaign to ensure people perceive they can participate
	**Environmental influences** ***Opportunity*** Invitation strategy Test design, location, opening hours Primary care endorsement Provider incentives Community endorsement Mass media	Education, persuasion Environmental restructuring Persuasion Incentivisation Modelling, education Education, persuasion	**Service provision/- environmental/social planning** Engaging and evidence-based invitation materials supporting access Provide a screening test accessible to all with additional support provided where necessary Future tests should be designed to optimise ease of use Include a primary care endorsement with invitation materials Provide incentives to Primary Care providers to support access **Communication/marketing** Identify key figures in the community to support access Engage with mass media to create narrative on supporting access
**Intention** Decided to act	**Participant influences** ***Capability*** Planning Self-efficacy **Environmental influences** ***Opportunity*** Invitation strategy Reminder strategy Patient navigation	Enablement Modelling Enablement, modelling Enablement/environmental restructuring Enablement, training	**Communication/marketing** Support people to make a plan about how, when and where they will do screening Support people to overcome barriers Support people to believe they are capable of doing screening **Service provision** Accessible information to support people to reach or complete screening e.g. maps and public transport suggestions, pictures to support self-completion, narratives of people who have participated Reminders to prompt action e.g. additional letters, calls, texts, verbal reminder if attending primary care Provide additional support where necessary to navigate people through the screening process
**Action** Acting	**Participant influences** ***Capability*** Positive screening experience **Environmental influences** ***Opportunity*** Positive screening experience Results framing	Enablement Environmental restructuring Environmental restructuring	**Communication/marketing** Support people to feel sense of mastery/accomplishment **Service provision** Supportive, timely, efficient screening experience **Communication/marketing** Supportive and accessible communication of results and follow up
Repeat	**Environmental influences** ***Opportunity*** Re-invitation	Environmental restructuring/ education, persuasion	**Service provision** Engaging and evidence-based invitation materials tailored to supporting repeated behaviour
**Stages of inaction**			
Decided not to screen	**Participant influences** ***Motivation*** Ensure knowledge of benefits and harms Address emotional beliefs and misconceptions ***Capability*** Other priorities	Education Enablement	**Communication/marketing** Support people to ensure they have made a good decision for them **Service provision** Ensure people have the necessary support to access screening

It will be important to establish the application of the I-SAM in low- and middle-income countries as the majority of the theoretical and empirical evidence is from high income countries. It is anticipated that the relative influence of the different components may differ between different income settings (Piyasena et al., 2019, Chidyaonga-Maseko et al., 2015).

## 5 Conclusions

The I-SAM proposes an integrated theoretical model to support our understanding of screening behaviour and to identify targets for intervention. It will be an iterative process to test and refine the I-SAM to ensure we capitalise on the benefits of theory-guided approaches, as they evolve. The I-SAM aligns with a proportionate universalism and precision medicine approach which is crucial as it is clear that our current ‘one-size-fits-all’ approach to screening is failing to engage equitably all sectors of the community. More targeted approaches are required to support those less likely to engage in screening such as people living in socioeconomic deprivation, ethnic minorities, people with comorbidities and learning disabilities and in different income settings. The I-SAM aims to provide an empirically and theory-driven approach to improve screening for all.

## CRediT authorship contribution statement

**Kathryn A. Robb:** Conceptualization, Methodology, Investigation, Writing - original draft, Writing - review & editing, Visualization.

## Declaration of Competing Interest

The authors declare that they have no known competing financial interests or personal relationships that could have appeared to influence the work reported in this paper.
